# Optimizing the Development of Space-Temporal Orientation in Physical Education and Sports Lessons for Students Aged 8–11 Years

**DOI:** 10.3390/children9091299

**Published:** 2022-08-27

**Authors:** Denisa-Mădălina Bălănean, Cristian Negrea, Eugen Bota, Simona Petracovschi, Bogdan Almăjan-Guță

**Affiliations:** Faculty of Physical Education and Sports, West University of Timișoara, 300223 Timișoara, Romania

**Keywords:** space–temporal orientation, general intelligence, psychomotricity, specific intervention

## Abstract

The purpose of this research was to analyze how we can improve the space–temporal orientation ability with the help of physical exercises in physical education and sports lessons. In total,148 children between the ages of 8 and 11 participated in this study (M = 9.70; SD = 0.79). They were subjected to three tests, which measured general intelligence (Raven Progressive Matrices) and space–temporal orientation skills (Piaget-Head test and Bender–Santucci test). The tests were carried out both in the pre-test and in the post-test period. In the case of participants in the experimental group, a specific program was applied for a period of 12 weeks. The results showed that general intelligence level was identified as a predictor of spatial–temporal orientation (beta = 0.17, t = 2.08, *p* = 0.03) but only for the Piaget-Head test. Similarly, no differences between children’s age groups were identified in any of the spatial–temporal orientation test scores. However, children in the “+9” age category had higher scores on the intelligence test compared to younger children (77.31 vs. 35.70). In conclusion, the intervention program had a positive effect on spatial orientation skills.

## 1. Introduction

### Conceptual Foundation

Psychomotricity is the ability of the human being to coordinate thought (analysis) and reaction (movement) in an optimal time, before a certain stimulus [1]. Until the beginning of the 20th century, psychomotricity was included in the field of psychology. As time passed, a new conception was born regarding the integral formation of the human being [2]. Thus, Suasnabas and his collaborators [3] refer to psychomotricity through its two factors: the psychic and the motor parts, both making possible the physical interaction with the mental one, forming a whole that develops emotions and knowledge [4]. This function plays a primary role in the stimulation and development of bodily capacities [5], positively influencing self-esteem and independence in a developmental process [6,7]. Therefore, adequate psychomotor development allows children to improve their balance, coordination [6], and space–time orientation, which is of great value for increasing intelligence and reasoning when performing motor actions [8].

Space–temporal structuring is the ability to perceive, to relate, to move, and to orientate, with everything that exists, with its education being the basis for obtaining adequate motor and affective development [9]. The development of space–temporal orientation is a process that takes place starting from an early age. It contributes at the same time to the cognitive, physical, and psychomotor growth of the child. It has an important contribution to the educational system as well [10,11,12]. A total or partial lack of space–temporal orientation can have repercussions not only in the teaching–learning process [13,14] but also in the social context, influencing cognitive development and the quality of life [15].

The development of spatiality is considered by some authors to be an evolutionary process which is progressively acquired until realization, throughout psychomotor development [8,9]. The importance of space–time perception is essential in human development in the sense that one can observe the shapes, structure, or compositions of objects as well as their location in relation to one’s own person or to those around him or her [16,17]. Thus, certain authors believe that [18] certain aspects must be considered for the development of spatial perception and structuring: orientation in space, appreciation of distances, appreciation of trajectories, and the space–time relationship. The authors of the previously mentioned study also believe that time is, in principle, closely related to space, being the duration that separates two successive spatial perceptions [18], constituting fundamental concepts in learning and cognitive development [19].

All children use spatial concepts in various areas of their lives as they are useful for reading, writing, running, playing, etc. However, this capacity is often not developed adequately [20]. There are cases when the child perceives space in relation to his/her body, it is clear to him/her that he/she is surrounded by peers and objects, but it is difficult for him/her to differentiate between them, to classify them in order or to identify a given distance [21]. Psychology states that this confusion occurs due to the inability to distinguish and perceive what they see, so they mainly use what they think [22]. Thus, there is a need for small children to establish a spatial order centered on their own person and, only then, on the connections they develop with the environment [23,24]. Simultaneously they develop the understanding of temporal notions, which helps them identify the past time and the future as well as moments of the day [25]. As a central aspect of development and evolution, an improvement in these skills helps children identify the spatial relationships between real objects and imaginary objects [26], recognizing space as much as they come to perceive and master it [27].

The general objective of the experimental study was to improve the spatial-temporal orientation ability of primary school students through specific exercise intervention. Given the general objective, the novel element is the testing of a possible predictor of spatial-temporal ability, namely the level of general intelligence. Currently, there are few studies that focus on these variables. Thus, obtaining results oriented in this direction could give value to this research.

## 2. Materials and Methods

### 2.1. Study Participants

The studied sample was composed of 148 children, students in the 3rd and 4th grades, of which 70 were boys and 78 were girls. The students were placed in groups using convenience sampling. The age range varied between 8.1 years and 11.9 years (M = 9.70; SD = 0.79). The children included in the study underwent an assessment for the level of intelligence. As a result, none of the students were excluded. Therefore, the number of participants in each group was equal. The experimental group included students from the 3rd grade and 4th grade (N = 74), aged between 8 years and 1 month, respectively 11 years and 9 months (M = 9.62, SD = 0.81). The control group included students from the 3rd grade and 4th grade (N = 74), aged between 8 years and 2 months and 11 years and 9 months, respectively (M = 9.78, SD = 0.77).

### 2.2. Objectives and Hypotheses of the Research

An experimental study was carried out, with the general objective of improving the ability of space–temporal orientation, with the help of intervention through specific exercises for primary school students.

**Hypotheses** **H1.** *It was assumed that the level of general intelligence influenced the ability of space–temporal orientation.*

**Hypotheses** **H2.** *There was a statistically significant difference between the age categories of the children in terms of the results on the assigned tests.*

### 2.3. Research Tools

The students were subjected to 3 psychological tests that determined general intelligence on the one hand and space–temporal orientation skills on the other.

#### 2.3.1. Raven Progressive Matrices

Raven’s Progressive Matrices Test is a test used in the field of psychology and psychopedagogy, which measures the general intelligence level of the subject. The main feature of this test is to encourage analytical reasoning, perception, and the ability to abstract. The students were given the first 4 series (from A to D), out of a total of 5, with 12 questions for each series (from A1 to D12), with these being organized according to progressive difficulty. The intellectual performance thus “measured” allowed the inclusion of the subject in one of the 5 different degrees (levels) of intelligence. For an explicit understanding of how to interpret results and convert test scores to percentiles, see Măłkiński and Măńdziuk [28]. It was decided to use this sample for 2 reasons:

1. One of the purposes of the study was to demonstrate that the sample consists of children of age-appropriate intelligence and the results will not be affected in any way by a possible low IQ of the students.

2. Children’s general intelligence was one of the dependent variables used to determine the degree of its association or influence on space–temporal orientation skills.

#### 2.3.2. Piaget-Head Space–Temporal Orientation Test

This test assesses the right–left spatial orientation of children between the ages of six and eleven [29]. The number of items the child must get right is 3 out of 3 at the age of 8, 6 out of 8 at the ages of 9 and 10, and 5 out of 6 at the age of 11. Successful trials are marked with +, unsuccessful ones with −, and spontaneously corrected trials with – +. The rating is done by adding up the successfully completed items.

#### 2.3.3. The Perceptual–Motor Test of Bender–Santucci Spatial Configuration

This test targeted the perceptual–motor function of spatial configuration by testing the ability of children between the ages of 5 and 15 to perceive spatial configurations and to make comparisons between the respective configurations, thus making a rendering of space and shape on paper [30]. The testing took place individually, with the necessary materials being represented by 8 cards. The rating was made considering certain criteria: the construction of the angles, the orientation of the figures or the component elements, and the position of the figures or the elements that make up the model.

### 2.4. Research Method

In the first stage, an agreement was made between Secondary School No. 24 from Timișoara, the Faculty of Physical Education and Sport from Timișoara, and the Timiș School Inspectorate, which allowed the study to continue. Then, a collaboration contract was drawn up between the author of the study and a specialized psychologist under whose guidance the psychological tests were applied and interpreted.

In the second stage, the consent of the parents or legal guardians was requested and obtained, and then the children participating in the study were tested with the “Raven Progressive Matrices” test, to determine that the included sample consisted of children with age-appropriate intelligence.

In the third stage, students were randomly assigned to one of the experimental or control groups. The investigated variable “space–temporal orientation” was measured in the pre-test phase, so that in the case of the participants in the experimental group, the intervention would be applied to this independent variable. The control group did not benefit from any kind of intervention or manipulation of the mentioned variable.

In the post-test phase, the variable “space–temporal orientation” was measured again, to determine if the intervention generated changes in the test results.

#### The Intervention Plan

The actual research took place between January and June 2022. With a frequency of 2 physical education and sports lessons per week, the students from the experimental group were given the intervention plan, which had as general themes and objectives the elements detailed in Table 1.

### 2.5. Statistical Analysis

The collected data were entered, processed, and analyzed using the IBM SPSS Statistics 20 program (IBM Corp, Armonk, NY, USA). Each hypothesis was tested using appropriate statistical techniques. To identify the predictor factor on the space–temporal orientation, linear regression was analyzed. Correlations between variables were checked using the Pearson test.

## 3. Results

### 3.1. Intelligence Test Results, with the Purpose of Including Children in the Sample

The score with the highest frequency was 75 (17), followed by 85 (frequency 11). This aspect showed us that the average of the students included in the sample had an above average intelligence level, taking into account the age, measured in years and months. With the help of this result, they intended to keep the level of intelligence under control, and the scores obtained were suitable for the inclusion of the children in the study.

### 3.2. Hypothesis Testing

**Hypothesis** **1.** *It was assumed that the level of general intelligence influenced the ability of space–temporal orientation.*

To test Hypothesis 1, a linear regression was used; however, before applying it, the correlation between the variables was verified by the Pearson test at a level of statistical significance of *p* less than 0.05. Thus, in Table 2 it can be seen that there is a statistically significant direct but weak relationship between IQ and spatial orientation for the Piaget-Head test (r = 0.17, *p* = 0.03).

After the application of the Pearson correlation we can expect IQ to be a predictive factor for spatial orientation measured by the Piaget-Head test.

- Linear regression with the dependent variable spatial orientation, measured with the Piaget-Head test

According to the results in Table 3 and Table 4, we could conclude that the model was successful (F(1,146) = 4.36, *p* = 0.03) and that the variance explained by IQ was 2.9% for spatial orientation measured by the Piaget-Head test. Furthermore, IQ could be considered a predictive factor for spatial orientation by the Piaget-Head test (beta = 0.17, t = 2.08, *p* = 0.03).

According to Table 5, we could see that the model was not successful (F(1.146) = 1.33, *p* = 0.25), and the rest of the interpretations did not make sense.

**Hypothesis** **2.** *There was a statistically significant difference between the age categories of the children in terms of the results on the assigned tests. Given that the variable “age” was continuous and the average age was 9.70, we transformed it into a categorical variable with the value 9. The new variable “age”can be seen in Table 6 and has the following composition:*

**Figure 1:**
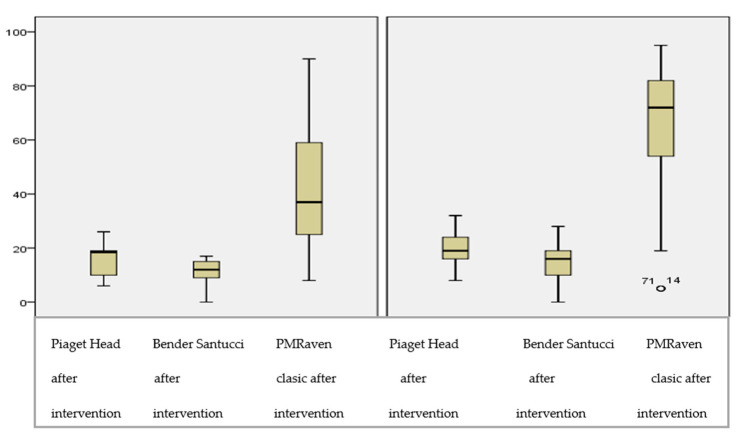
Age range of participants (<9 years/>9 years).

Regarding the difference between the two age categories, the results of the spatial orientation tests did not reveal any statistically significant index according to the month and year of birth, but a statistically significant difference was recorded at the IQ level (M − W = 302.00, *p* = 0.003). Thus, children older than 9 years had a higher IQ (77.31 vs. 35.70), which can be seen in Figure 1.

In conclusion, Table 7 illustrates the situation of the hypotheses.

## 4. Discussion

The present study had as its general objective the improvement of the ability of spatial orientation in children between the ages of 8 and 11. Being an experimental study in which the proposed sample was divided into two groups (experiment and control) by convenience, one of the objectives was to keep under control the intelligence of the children included, before any other approach. Thus, the results of the Raven Progressive Matrices testing showed us that most of the included students had an above average level of intelligence, taking into account the age, measured in years and months (between 8 and 11 years). At the same time, general intelligence was identified as a predictive factor for spatial orientation, but only for the Piaget-Head test. In the same context, spatial ability, which refers to “the location of objects, their shapes, the relationship between them and the paths they take as they move” [31], has long been recognized as an ability partially independent of general intelligence [32,33]. In addition to being distinct from other cognitive abilities, spatial thinking itself has often been conceptualized in a multidimensional manner, consisting of several separate but related abilities. Thus, a result that supports the partial confirmation of our first hypothesis is a relatively recent study which discovered a relationship between certain spatial components involved in a puzzle game and the results of a preschool and primary intelligence test (WPPSI) [34]. Even though a direct relationship between general intelligence and space–temporal orientation has not been found in the studies conducted in recent years, certain studies did identify a relationship between executive function and spatial abilities [35,36]. However, we cannot affirm following these researches that the two components can be predictive factors. Thus, the relationship between the two is more assumed, studies being directed to the spatial components related to performance in mathematics and geometry [37,38,39]. Similarly, we do not know whether these assumed relationships are variable with age or remain stable across the lifespan. Although most children of this age are developing many of the cognitive skills necessary for successful spatial orientation [40,41], there is increased neural activity in areas of the brain associated with visual–spatial processing compared to young adults [42]. Thus, the ability to orient and navigate is a cognitive process that undergoes a maturation with the progression of skills and strategies during a large period of the childhood. These findings support that as children mature, they increase and refine their proficiency in visual and spatial skills, increasing network connectivity and enabling the successful use of spatial orientation strategies.

Verification of Hypothesis 2 confirmed the fact that students in the age category >9 years (10/11) recorded higher scores in terms of IQ, compared to the category ≤9 (8/9 years).

This result was consistent with two other studies in the field of psychology, in which the younger age groups showed a lack of maturation of the functionality of the prefrontal regions, which is involved in these types of tasks [43,44]. However, studies of brain development have shown that around 8 to 9 years of age, significant structural and functional changes [45] affect the whole brain and gray matter volume [46], synaptic pruning processes [47], and functional connectivity [48,49,50]. Furthermore, studies have shown that around the age of 10, there is a significant increase in cortical thickness in the parietal and frontal areas as well as in higher-order cortical areas such as the dorsolateral prefrontal cortex and the cingulate cortex [51,52]. Therefore, it is possible that such changes in brain development support a greater development of cognitive potential in the 8- to 10-year-old groups than in the younger ones, which is not consistent with the result of our study.

This study might have some limitations. First, the study sample consisted of children between the ages of 8 and 11, so the results may not be suitable for the entire general population. Additionally, the findings of the present study apply to subjects attending academic institutions in the urban region, which may be inappropriate for children in rural regions or who have dropped out of school. Second, although the variable “general intelligence” was kept under control, the results may have been influenced by other factors, such as the extracurricular activities that many of the subjects attended during the intervention period. At the same time, the large number of participating students could add value to the study.

## 5. Conclusions

The intervention program through specific exercises had the expected effect. Moreover, it was determined that the level of intelligence, as measured by percentiles, was a predictive factor only for the first spatial orientation test but not for the second. This reason might have been due to the content of the Santucci test, which relied to a greater or lesser extent on graphic qualities and imitability, aspects for which we do not know whether they are associated with children’s IQ. This finding can lead to a new direction of research in which a possible connection between graphic qualities and the ability of space–temporal orientation and the level of general intelligence can be highlighted.

That is precisely why, in order to further explore the influence of some intervention programs on space–temporal orientation, it is essential to investigate its impact on several components, keeping under control factors related to age, gender, social environment, and level of general intelligence.

## Figures and Tables

**Table 1:** General themes and objectives.

Lesson	General Theme and Objective
Space Orientation	T: Body References (left side, right side): Knowing and identifying the right side and left side of the body
Space–Temporal Orientation	T: Recognition and operation with spatial and space–temporal notions G.O: Formation, recognition, and operation with space and space–temporal notions of one’s own body (left side, right side). Interval duration perception
Space Orientation	T: Recognition and operation with space notions of one’s own body, in relation to surrounding objects G.O: Formation, recognition, and operation with space notions of one’s own body, in relation to surrounding objects (left side, right side)
Space–Temporal Orientation	T: Recognition and estimation of distances G.O: Recognition and estimation of distances, in due time. Left side/right side reminder
Space Orientation	T: Establishing the direction/position of objects G.O: Establishing the direction/position of objects in relation to one’s own person but also to each other, as well as imitating certain actions
Space–Temporal Orientation	T: Knowledge of direction and axes G.O: Establishing the direction/position of objects in relation to one’s own person, as well as orientation in space and time
Space Orientation	T: Working with space notions G.O: Recognizing and operating with space notions located in space near and far from one’s own body. Linear orientation
Space–Temporal Orientation	T: Knowledge of two-dimensional space G.O: Recognition and estimation of distances (quantities) in two-dimensional space
Space Orientation	T: Knowledge of spatial concepts G.O: Identifying the notions of: “outside, inside/full, empty”
Space–Temporal Orientation	T: Strengthening the estimation of distances G.O: Recognition and estimation of distances. Interval duration perception
Space Orientation	T: Knowledge of spatial concepts G.O: Identifying the notions of “above and below”
Space–Temporal Orientation	T: Strengthening the estimation of distances G.O: Recognition and estimation of distances (quantities) in two-dimensional space and time
Space Orientation	T: Knowledge of spatial concepts G.O: Recognition and estimation of distances (length-width, near-far)
Space–Temporal Orientation	T: Relating to the environment G.O: Identifying the position of objects in relation to one’s own person and orientation in space and time
Space Orientation	T: Knowing dimensions G.O: Knowledge of the notion of size and sensory-motor practice: organizing objects of the same nature according to size criteria: big-small, long-short, tall-short, etc., presented or not in the perceptual field
Space–Temporal Orientation	T: Strengthening the estimation of distances, in relation to time G.O: Recognition and estimation of distances, correlated with time
Space Orientation	T: Consolidation of spatial concepts G.O: Formation, recognition and use of spatial notions located in near and far space in relation to one’s own body and in relation to others. Practicing mathematical calculations
Space–Temporal Orientation	T: Knowledge of direction and axes G.O: Establishing the direction/position of objects in relation to one’s own person, but also to each other
Space Orientation	T: Consolidation of spatial concepts G.O: Recognizing and estimating some distances (quantities) in two-dimensional space, as well as operating with the notions of “up-down, forward and backward”
Space–Temporal Orientation	T: Relating to the environment G.O: Identifying one’s own body and other objects in space and time
Space Orientation	T: Strengthening the estimation of distances and direction of movement G.O: Recognizing and estimating distances, as well as determining direction
Space–Temporal Orientation	T: Consolidation of spatial concepts G.O: Formation, recognition and use of spatial notions of one’s own body and of other people (left-right, above-below, forward-backward, up-down, etc.).

**Table 2:** Correlations between variables.

Correlations				
		Piaget-Head Test after the Intervention	Bender–Santucci Test after the Intervention	Raven Spm Classical Progressive Matrices
Piaget-Head test after the intervention	Pearson Correlation	1	0.051	0.170 *
	Sig. (2-tailed)		0.540	0.039
	N	148	148	148
Bender–Santucci test after the intervention	Pearson Correlation	0.051	1	0.095
	Sig. (2-tailed)	0.540		0.250
	N	148	148	148
Raven Spm Classical Progressive Matrices	Pearson Correlation	0.170 *	0.095	1
	Sig. (2-tailed)	0.039	0.250	
	N	148	148	148

* Correlation is significant at the 0.05 level (2-tailed).

**Table 3:** Variance explained in Piaget-Head spatial orientation by IQ.

Model	R	R Square	Adjusted R Square	Std. Error of the Estimate
1	0.170 ^a^	0.029	0.022	5.53416

^a^ Predictors: (constant), Raven Spm Classical Progressive Matrices.

**Table 4:** Significant coefficients of the model with the dependent variable, spatial orientation, for the Piaget-Head test.

Model		Unstandardized Coefficients		Standardized Coefficients			95.0% Confidence Interval for B	
		B	Std. Error	Beta	T	Sig.	Lower Bound	Upper Bound
1	(Constant)	16.921	1.444		11.716	0.000	14.067	19.776
	Raven Spm Classical Progressive Matrices	0.044	0.021	0.170	2.088	0.039	0.002	0.086

Linear regression with the dependent variable, spatial orientation, measured with the Bender–Santucci test.

**Table 5:** Explained variance of Bender–Santucci spatial orientation by IQ.

Model	R	R Square	Adjusted R Square
1	0.095 ^a^	0.009	0.002

^a^ Predictors: (constant), Bender-Santucci test.

**Table 6:** Age range.

Age per Range			
		Frequency	Percent
Valid	Age < 9 years	10	6.8
	Age > 9 years	138	93.2
	Total	148	100.0

**Table 7:** Situation of the hypotheses.

Hypothesis No.	Status
I1.	Partially Accepted
I2.	Partially Accepted

## Data Availability

Data can be available for consultation when requested from the corresponding author.
